# Outcomes of integrated surgical wound treatment mode based on tibial transverse transport for diabetic foot wound

**DOI:** 10.3389/fsurg.2022.1051366

**Published:** 2023-01-16

**Authors:** Shusen Chang, Fang Zhang, Wei Chen, Jian Zhou, Kaiyu Nie, Chengliang Deng, Zairong Wei

**Affiliations:** ^1^ Department of Burn and Plastic Surgery, Affiliated Hospital of Zunyi Medical University, Zunyi, China; ^2^ The Collaborative Innovation Center of Tissue Damage Repair and Regeneration Medicine, Zunyi Medical University, Zunyi, China

**Keywords:** integrated surgical wound treatment, diabetic foot ulcer, tibial transverse transport, antibiotic bone cement, vacuum sealing drainage

## Abstract

**Background:**

Diabetic foot ulcer (DFU) is frequently difficult to heal and finally leads to amputation, resulting in high mortality rate in diabetic patients. To date, effective and optimal therapies are still lacking. This study aims to investigate the efficacy of integrated surgical wound treatment (ISWT) mode on diabetic foot wound.

**Methods:**

From January 2021 to December 2021, 13 diabetic foot patients with Wagner grade 3 to 4 were treated with ISWT mode, which combined TTT technique with debridement, induced membrane technique, vacuum sealing drainage (VSD) technique and skin grafting technique. The time of wound healing, the skin temperature at midpoint of dorsum of affected foot (T), visual analogue scale (VAS) score and ankle-brachial index (ABI) was measured before and after surgery. CTA examination of the lower extremity arteries was performed at the end of the cortex transport to evaluate the small arteriolar formation of the lower extremity. The complications occurred in each patient were recorded.

**Results:**

13 patients with age ranging from 45 to 66 years were followed up for 3 to 13 months. All patients healed completely without amputation being performed, no serious complications were found except for one case of nail channel infection. The mean healing time was 25.8 ± 7.8 days, with a range of 17 to 39 days. The mean time of carrying external fixation scaffolds and resuming walking was 71.8 ± 10.0 and 30.8 ± 9.1 days, with a range of 56 to 91 days and 18 to 45 days, respectively. The skin temperature at midpoint of dorsum of affected foot (T), VAS and ABI was all improved significantly at 3 months after surgery. Furthermore, CTA examination showed an increase in the number of lower extremity arteries and a thickening in the size of small arteriolar compared with those of pre-operative, and the collateral circulation of lower extremity was established and interweaved into a network.

**Conclusion:**

Integrated surgical treatment of diabetic foot wound can achieve satisfactory clinical results.

## Introduction

Diabetic foot ulcer (DFU) is one of the most serious chronic complications in patients with diabetes, bringing heavy economic burdens for family and public health system. DFU usually manifests as distal ankle skin infection, ulcer and/or deep tissue destruction caused by complex factors including lower extremity vascular disease and local nerve abnormalities, characterized with high morbidity, high disability rate, high mortality rate and high cost (1–3). Epidemiological studies show that the prevalence of DFU is approximately 6.3% worldwide, ranging from 1.5% to 16.6%, with great difference between different countries and regions (4, 5). Severe DFU is usually associated with a range of local complications such as gangrene, deep and large ulcers, osteomyelitis, infection and chronic wounds, which will finally lead to amputation. It's reported that 90% DFU patients with Wagner grade 3 to 5 have to be amputated eventually, with the 5-year mortality rate reaching 25% to 50% after amputation (6, 7). Furthermore, even ulcers healed after conventional treatment, the recurrence rate is still as high as 40% within 1 year, 60% within 3 years and 65% within 5 years, respectively (8). Therefore, novel and effective therapeutic methods are urgently needed to promote DFU healing, improve limb salvage rate and reduce recurrence.

Tibial cortex transverse transport (TTT) technique is a new surgical procedure for treating severe chronic limb ischemic diseases such as DFU. This technique is based on the stress-tension principle established by Ilizarov, through which tension promotes the growth and regeneration of bone and soft tissue (9–12). A series of animal experiments and studies have shown that distraction osteogenesis is often associated with angiogenesis of the surrounding tissues. In the process of bone transport, a large number of new blood vessels are generated around the transported bone block as well as the distal of the lower extremities, leading to the formation of collateral circulation (9, 12, 13), which results in the redistribution of blood flow, thus promoting local blood supply and venous return, restoring the microcirculation of the lower extremity and thereby promoting the healing of foot ulcers, avoiding amputation and reducing the risk of ulcers recurrence (13–16).

The traditional treatment of diabetic foot ulcers is usually multi-disciplinary team (MDT) treatment, which requires the participation of physicians from interventional, plastic, orthopedic and other departments. In the current study, we proposed the integrated surgical wound treatment (ISWT) mode, a mode that integrates multiple surgical techniques for wound treatment, for the treatment of DFU according to the domestic medical situation and the experience of our department. By the application of ISWT mode, the patients can get more reasonable and effective treatment, thus improving the efficiency of diagnosis, and improve the diagnosis and treatment ability of the team. Based on TTT technique, ISWT integrates debridement, induced membrane technique, vacuum sealing drainage (VSD) technique and skin grafting technique to treat diabetic foot ulcers, and has achieved good clinical results. We reported here the exciting results of this treatment mode.

## Materials and methods

### Patients

The study included DFU patients with Wagner grade 3 to 4 treated at Affiliated Hospital of Zunyi Medical University who received the surgery by the same surgeon from January 2021 to December 2021.

### Clinical and imaging evaluation

Wagner Classification System was used to evaluate the severity of the ulcer (17) and the surface area of the diabetic ulcer and the duration of DFU of each patient were shown in supplementary Table 1. To identify the pathogen and its antibiotic sensitivity, a bacterial culture of wound secretions was performed after hospitalization. Moreover, each patient has an x-ray examination of the affected limb to assess the continuity of the tibia and detect osteomyelitis. All patients have a computed tomography angiography (CTA) examination of the lower extremity, which showed occlusion or stenosis of the arteries below the knee joint, with at least one artery patently reaching the level of the ankle joint. The inclusion criteria were as follows: (1) ages of >18 and <80 years; (2) with diabetic foot of Wagner grade 3 to 4; and (3) the arteries below the knee had varying extent of stenosis or occlusion, and at least one artery (anterior tibial, posterior tibial, and fibular artery) was unobstructed to the ankle plane with CTA. Criteria for exclusion were: (1) severe peripheral vascular diseases were unable to receive vascular reconstruction; (2) coagulopathy, mental illness or severe malnutrition (3) pregnancy; (4) patients with a history of cardiac failure, multiple organ dysfunction, cancer, or renal failure; (5) patients treated with corticosteroids, immunosuppressive drugs, and/or chemotherapy; (6) patients wore inability to tolerate surgery and anesthesia.

**Table 1:** Outcomes of patients.

Item	Value
Carrying external fixation scaffolds (days)*	71.8 ± 10.0
Resuming walking (days)*	30.8 ± 9.1
Full closure (days)*	25.8 ± 7.8
Amputation (number)	0
Complication (number)	1

* Values are expressed as mean ± standard deviation for continuous variables.

### Treatment methods

#### Pre-operation

The blood glucose of each patient was controlled well before operation, with fasting blood glucose (FBG) < 7.2 mmol/L and postprandial blood glucose (PBG) < 11 mmol/L. For all patients with infection, empiric single antibiotic therapy was used at first, and sensitive antibiotic therapy was given after the antibiotic sensitivity was confirmed. Patients with underlying diseases and internal environment disorder were given symptomatic treatment to improve the general condition firstly. If the wound is severely infected, an emergency debridement was given.

#### Surgical techniques

##### Debridement and covering wound with bone cement

After general anesthesia, the wound was alternately washed with hydrogen peroxide, normal saline, and light iodophor. After disinfecting and laying sterile towel, debridement was performed to remove necrotic tissue (including inactive soft tissue, adipose tissue, tendon and infected necrotic bone) from the wound. Then, washing the wound again with the liquid described above and stopping the bleeding thoroughly. Subsequently, using German heraeus antibiotic bone cement (gentamicin: bone cement = 0.5: 40) covered the wound according to the pre-operative antibiotic sensitivity results and 1g vancomycin powder was added into the bone cement for the patients who were sensitive to vancomycin. Add stock solution to the bone cement and mix well, then cover the wound and fill the cavity when the bone cement mass was like plasticine. The thickness of the bone cement was about 2–3 mm. The bone cement was drilled to facilitate drainage. Rinse with normal saline to cool the bone cement down. Subsequently, 4# silk thread was used to secure the bone cement and sterile dressing was used for bandage. Then, after dressing change and the wound being evaluated without infection and necrosis, the patients were discharged from hospital. 2–3 weeks later, the patients were called back for the next surgery.

##### Debridement and VSD negative pressure drainage

After general anesthesia, the bone cement was removed and the wound was washed with normal saline. After disinfecting and laying sterile towel, remove the necrotic tissue in the wound bed and around the wound. Trim the skin margin without injuring the induced membrane that is induced by the bone cement. After the normal saline was washed again, VSD negative pressure drainage was placed. After 3–5 days of drainage, the VSD negative pressure drainage was removed and TTT surgery was performed.

##### Tibial cortex transverse transport (TTT) and skin grafting

After general anesthesia, VSD negative pressure drainage was removed and the wound was washed with normal saline and light iodophor. After disinfecting and laying sterile towel, sterile rubber gloves were used to cover the wound so that the area that were going to perform TTT surgery could be protected from cross contamination. Firstly, TTT surgery was performed. Briefly, a bone block of 5–7 cm in length and 1.5–2 cm in width was designed in the upper-middle segment of the tibia. Then, a 7–10 cm arcuate incision was made in the medial side of the tibia and the skin was dissected until the periosteum without separating the subcutaneous tissue. Suture the periosteum to the skin to avoid separation. The skin, together with the periosteum, was peeled laterally and turned up to the osteotomy position. Subsequently, drill holes with a 1.8-mm drill bit under the guidance of a 3-hole osteotomy apparatus and cut off the longitudinal cortical bone on both sides of the bone block using a thin bone knife. Then, two 4-mm bone traction needles were inserted into the bone block and external fixation scaffolds were installed. After that, cut off the transverse cortical bone on both sides of the bone block. Then, the bone block was pulled outward to ensure that the osteotomy was complete and was then returned to the original position. After rinsing with normal saline, the periosteum and subcutaneous layer were sutured with 3–0 absorbable thread, and the skin was sutured with 0# silk thread. Then cover the operative area with sterile dressing. Secondly, a thin intermediate thickness free skin graft was taken from the thigh and punched for later use. Thereafter, the wound was debrided and rinsed with normal saline. Then, the free skin graft was transplanted to repair the wound and covered with VSD negative pressure drainage. Finally, cover the operative area with sterile dressing.

#### Post-operation and cortex transport

The blood glucose of each patient was closely monitored and kept at a level of FBG < 7.2 mmol/L and PBG < 11 mmol/L. All patients were given anti-infective therapy for 2–3 days after operation. The patients were discharged when the bone cement placement surgery was done and the wound infection was controlled well. All patients were required to return to the hospital 20 days later for the next operation. After the VSD negative pressure drainage surgery, 1000 ml normal saline was used daily for wound cavity rinsing, and the negative pressure was removed 4–6 days after surgery for subsequent TTT surgery combined with free skin grafting. 4–5days after TTT and free skin grafting, the bone block was transported outward at a rate of 1 mm per day, which was completed in four times. One week after transportation, x-ray examination was performed again to determine the location of bone block. After transporting for 14 mm, the bone block was maintained *in situ* for 3 days and then transported back at the same speed until it returned to the original position, and x-ray examination was performed to determine the position of the bone block. 2 months after resetting the bone block, x-ray examination was performed again to confirm that the bone window was healed and the external fixator was then removed. Notably, the VSD was removed 5–7 days after the free skin grafting, and the patients was discharged after the dressing being changed for 2–3 times and the grafted free skin being assessed to be survived.

All the procedures had been approved by the Ethics Committee of the Affiliated Hospital of the Zunyi Medical University, which were in line with the principles outlined in the Declaration of Helsinki (No. KLL-2022–639).

### Outcomes

The primary outcomes included the time of wound healing, the skin temperature at midpoint of dorsum of affected foot (T), visual analogue scale (VAS) score and ankle-brachial index (ABI). CTA examination of the lower extremity arteries was performed at the end of the cortex transport to evaluate the small arteriolar formation of the lower extremity. The complications occurred in each patient were recorded.

### Statistical analysis

All analyses were performed with SPSS 23.0 software. Results were presented as means ± standard deviation (SD). Comparisons between pre-operation and post-operation were analyzed by Student's *t*-test. *p* < 0.05 was considered to be statistically significant.

## Results

**Figure 1:**
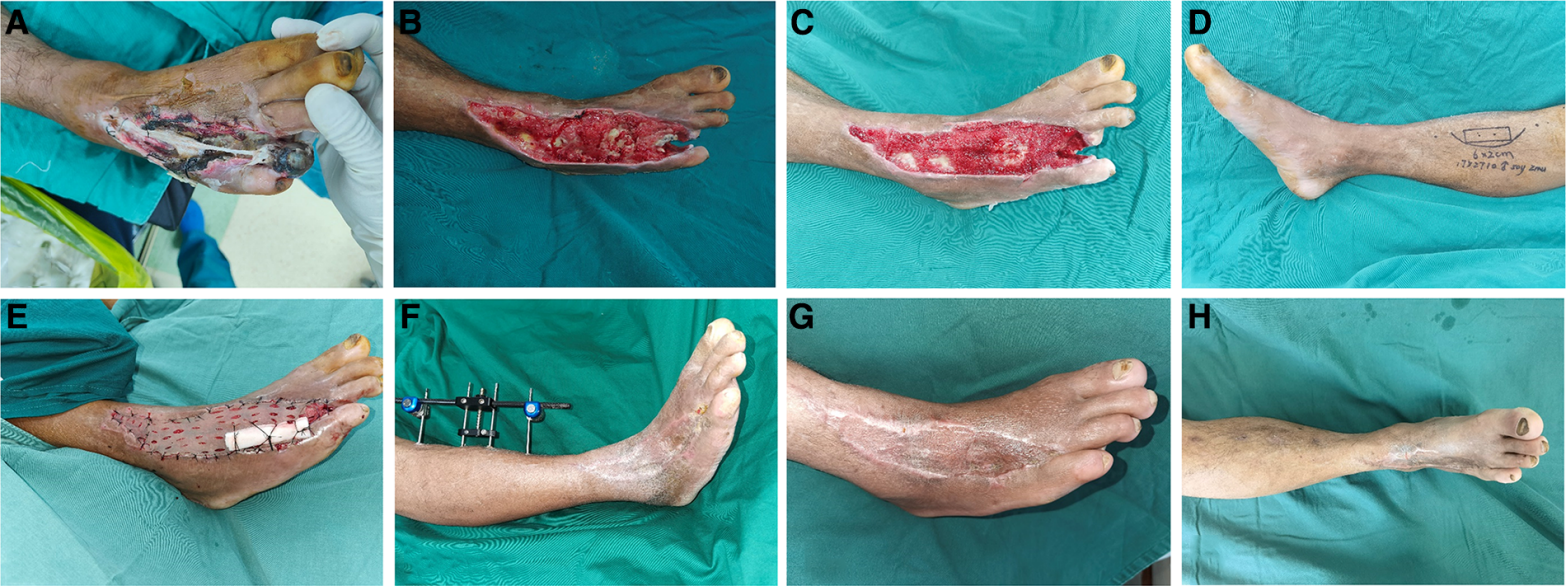
Typical case 1. (**A**) Pre-operative wound appearance; (**B**) Appearance of the wound after the bone cement removal; (**C**) The wound appearance after VSD aspiration; (**D**) Surgical design of TTT; (**E**) Immediate appearance after skin grafting; (**F**) Appearance after bone block being transported back; (**G,H**) Appearance of the operative area 6 months after TTT.

**Figure 2:**
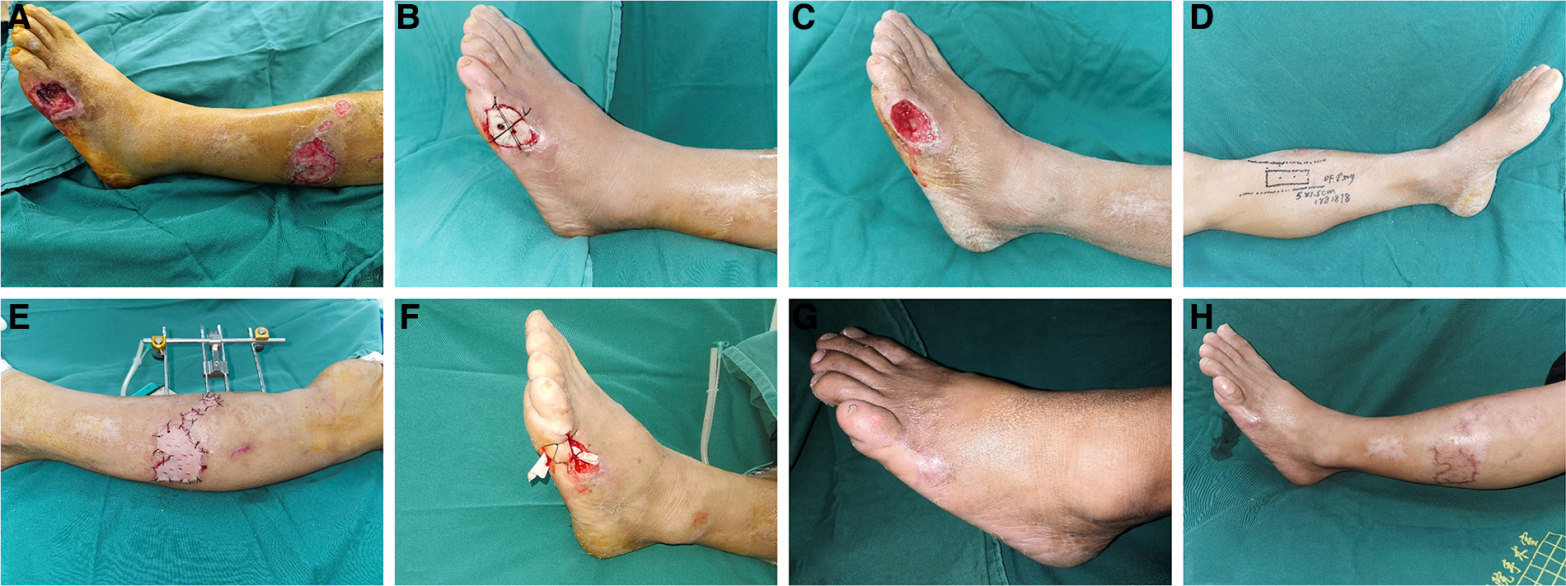
Typical case 2. After debridement, the wound on the foot was treat with bone cement and the wound on the lateral crural region was treated with VSD, and then TTT surgery was performed. (**A**) Pre-operative wound appearance; (**B**) Immediate appearance of the wound after covering with bone cement; (**C**) Appearance of the wound after the bone cement removal; (**D**) Surgical design of TTT; (**E**) Immediate appearance after skin grafting; (**F**) The wound on the foot was sutured directly with open drainage; (**G,H**) Appearance of the operative area 3 months after TTT.

**Figure 3:**
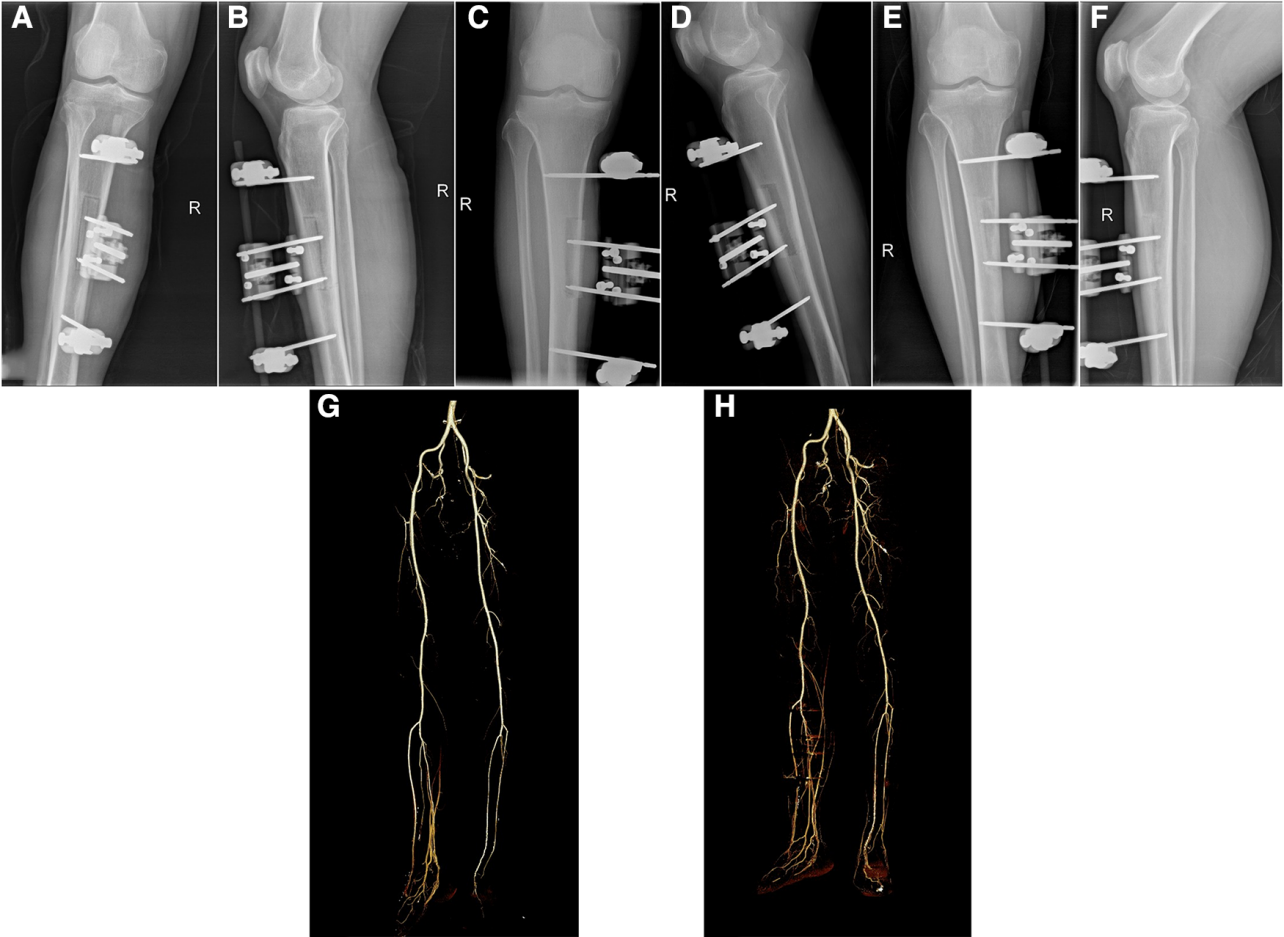
Imaging evaluation of typical case 1. (**A,B**) Immediate x-ray image after TTT surgery; (**C,D**) X-ray image at 12 days after bone cortex transport; (**E,F**) X-ray image at 2 months after TTT surgery; (**G**) Pre-operative image of CTA; (**H**) Image of CTA at 2 months after TTT surgery.

A total of 13 patients were included, with age ranging from 45 to 66 years. There were 9 males and 4 females, with a duration of diabetes for 2 to 11years. 5 patients got left feet affected while the other 8 patients got right feet affected. All patients had different degrees of infection symptoms, including redness, ulceration and necrosis. Among them, 8 cases had diabetic foot of Wagner grade 3 and 5 cases had Wagner grade 4. Each patient received different combined surgical methods based on TTT according to the actual situation of the wound, among which five patients received the method of TTT combined with bone cement, and seven patients received the method of TTT combined with bone cement and VSD and skin grafting (Supplementary Table S1). Patients were followed up for 3 to 13 months after surgery. Finally, the wounds of all the patients included in the current study healed completely. Limb salvage was achieved in all patients and no amputation was performed. Only one case of pin-site infection was found in the current study and healed after the pin being removed and dressing change for 2 weeks. No other complications occurred during follow-up period. No fractures at bone transport site, incision infection, cutaneous necrosis, bone nonunion, osteomyelitis or ulcer recurrence was observed (Table 1 and Supplementary Table S1). Two typical cases were shown in Figures 1, 2. Figure 3 showed the location of bone block during the cortex transport process.

In the current study, the mean healing time was 25.8 ± 7.8 days. The mean time of carrying external fixation scaffolds and resuming walking was 71.8 ± 10.0 and 30.8 ± 9.1 days, with a range of 56 to 91 days and 18 to 45 days, respectively (Table 1 and Supplementary Table S1). The skin temperature at midpoint of dorsum of affected foot (T), VAS and ABI was all improved significantly at 3 months after surgery, with the post-operative T increased by approximately 2.6 °C and ABI increased by about 0.2 compared with the values of pre-operative (Table 2 and Supplementary Table S2). Furthermore, CTA examination of the lower extremity arteries showed an increase in the number of lower extremity arteries and a thickening in the size of small arteriolar compared with those of pre-operative. The collateral circulation of lower extremity was established and interweaved into a network (Figures 3G,H), indicating that integrated surgical wound treatment based on TTT improves the blood supply of the distal limb.

**Table 2:** T, ABI and VAS of patients.

Test	Pre-operation	3 months post-operation	*t* value	*p* value
T (°C)	27.3 ± 1.5	29.9 ± 1.9	−12.04	<0.001
ABI	0.7 ± 0.2	0.9 ± 0.1	−10.43	<0.001
VAS	5.2 ± 1.2	1.4 ± 1.0	18.75	<0.001

## Discussion

The occurrence of DFU was related to multiple factors. Vasculopathy, neuropathy and immune system disorder all contribute to the pathophysiology of DFU. The persistent hyperglycemia of diabetes leads to lesions in the peripheral arteries of the foot, which results to foot microcirculation disorders and local tissue ischemia (18). Furthermore, DFU is often complicated with lower local immunity, which results in easy occurrence and difficult control of infection (7). The infections of DFU are mainly divided into two categories: one manifests as acute infection, which is often due to local skin rupture leading to spread of infection along the tendon sheath, myofascial space, and plantar aponeurosis, characterized with acute onset and rapid progression; the another one is characterized with chronic ischemia, necrosis and gangrene of toes (2, 8). Infection control is one of the critical steps for the treatment of DFU and it is particularly important to prevent sepsis caused by infection from causing body damage. Then, after the infection is controlled and the internal environment is stabilized, reconstruct the microcirculation of the limb and repair the wound through a certain way. In the current study, we controlled infection through debridement, antibiotic bone cement covering, VSD drainage firstly, then improve the blood supply of lower limb and repair the DFU wound by TTT and skin grafting, obtaining the good effect. As a result, we put forward the concept of integrated surgical wound treatment (ISWT) for the treatment of DFU and proved that ISWT based on TTT has excellent outcomes in clinical application.

Intravenous antibiotics are commonly used to treat infectious diseases, however, this approach often makes it difficult to achieve the required effective concentration of antibiotics in the infected lesion, resulting in the difficulty of adequate and effective disinfection, as well as the emergence of drug-resistant bacteria (19, 20). It is reported that antibiotic bone cement is able to release local antibiotics slowly and persistently and has been an effective tool for the treatment of local infection. The vancomycin and gentamicin contained in bone cement have good thermal stability and can achieve high local drug concentration and lasting elution rate, thus have good effect on controlling infection and reducing the emergence of resistant bacteria. Moreover, compared with systemic antibiotics, bone cement can reduce the adverse reactions such as liver and kidney toxicity. Importantly, bone cement can also isolate the wound from the outside environment and fill the cavity effectively, so that reduce bacterial colonization. Expect for effective anti-infection, bone cement can also form induced-membrane, which can secrete vascular endothelial growth factor (VEGF) and other angiogenesis related factors, thus promoting wound healing (9, 11, 13).

VSD technique has been widely used for the treatment of various wound (21, 22). Under the stimulation of negative pressure, the material is fully contacted with the wound, creating local tension through the pores between the material, so as to achieve comprehensive, thorough and sufficient drainage and reduce the absorption of toxins. Moreover, the pumping effect produced by VSD can significantly increase the blood flow velocity of microcirculation in the wound, dilate microvessels, improve vascular permeability and increase the number of capillaries, as well as stimulate the division and proliferation of wound repair cells (23). Additionally, VSD can timely remove exudate in the wounds, thus reducing tissue compression and microcirculation resistance, improving blood flow velocity, and promoting the growth of granulation tissue in the wounds. At the same time, it can accelerate the circulation of lymphatic fluid, thereby promoting the alleviation of edema degree (24, 25). The use of VSD creates a closed and humid environment relatively isolated from the outside environment, which helps immune cells to better play their functions and promotes the infiltration of polymorphonuclear leukocytes and T cells, as well as reducing the colonization of bacteria and preventing infection (26, 27). In our study, the recommended value of negative pressure was −80–−60 mmHg, the negative pressure was continuously existed within 48 h after operation, followed by intermittent negative pressure mode, that is, negative pressure suction for 5 min and pause for 2 min.

TTT technique was firstly used by Chinese orthopedic surgeons in the treatment of ischemic diseases of lower limbs, such as diabetic foot and thromboangiitis obliterans, and had achieved certain effects (13, 15, 16). TTT technique is based on the tension-stress principle. By giving certain stress to the callus and surrounding soft tissues, tissue regeneration ability is activated and strengthened, peripheral angiogenesis and blood flow redistribution are promoted, so as to increase local blood supply and improve microcirculation. Studies of multi-center and small volume showed that TTT technique can induce the regeneration of microvascular network of lower limbs, promote wound healing, and reduce the overall risk of diabetic foot (28). Recently, Yang et al. confirmed that TTT technique can accelerate the healing speed and improve the healing quality of wound by accelerating local blood flow, promoting neovascularization and regulating local inflammation in wounds (13). However, the treatment with TTT alone has a long healing time, and there is a risk of wound reinfection.

Using TTT technique or bone cement-induced membrane technique alone requires a long time of dressing change, accompanied by other factors such as the economic level of patients, living conditions, and different dressing change levels in different regions, leading to a long cycle of treatment, difficulty for wound healing, and even deterioration of the wound. As a result, the cost of treatment is further increased. Therefore, we integrated these techniques and combined the advantages of TTT and antibiotic bone cement in the treatment of diabetic foot wounds, and achieved excellent clinical results. By the combination of bone cement and TTT, some relatively small ulcers could be cured after two weeks of TTT (Figure 2). For patients with large ulcers, TTT combined with bone cement, VSD and skin grafting can achieve better clinical results, shorten the treatment period and improve the quality of wound healing (Figure 1). It is worth noting that debridement must be complete, and pre-operative MRI examination should be performed to confirm the extent of infection when necessary. When using VSD, the infected wound should be turned into a clean wound, and the bone cement induced-membrane should not be destroyed. We suggested that the application time of TTT technique should be after treating with bone cement and no bacterial growth in the wound. After getting effective control of infection, the risk of surgery and complications will be significantly reduced, and the patient's recovery will be more conducive. Furthermore, good patient compliance is required during the treatment.

In conclusion, on the basis of first-stage thorough debridement, we provided good wound bed preparation for skin grafting by combining the properties of antibiotic bone cement with the advantages of VSD. Meanwhile, the use of TTT technique induced the regeneration of microcirculation in lower limbs, promoted wound healing, improved the tissue quality and increased the anti-infection ability of tissue after wound healing, which has achieved good clinical effects. However, the fact that should not be ignored is that TTT technique has a long time of carrying external fixation scaffolds, leading to inconvenience to patients. Moreover, the number of cases included in this study is small and the follow-up time is short, and the long-term efficacy needs further observation and study and it is necessary to conduct more large-sample studies in the future. In addition, further studies are needed to determine whether there are differences in tissue quality and long-term recurrence rate after wound healing between ISWT and one or two methods alone in the treatment of diabetic foot.

## Data Availability

The original contributions presented in the study are included in the article/**Supplementary Material**, further inquiries can be directed to the corresponding author/s.
