# Supplementary material for: Outcomes of integrated surgical wound treatment mode based on tibial transverse transport for diabetic foot wound
Source: Front Surg. 2023 Jan 16;9:1051366. doi: 10.3389/fsurg.2022.1051366 (PMC9885215; doi:10.3389/fsurg.2022.1051366)
Supplement: Supplementary file 1 [file Table1.docx]

| **Patient** | **Age** | **Gender** | **Wagner classification** | **Surgical method** | **Duration of DFU (days)** | **Wound size (cm^2^)** | **Full closure (days)** | **Resuming walking (days)** | **Carrying external fixation (days)** | **Complications** |
| --- | --- | --- | --- | --- | --- | --- | --- | --- | --- | --- |
| 1 | 45 | Male | 3 | TTT + bone cement | 12 | 12 | 18 | 20 | 56 | None |
| 2 | 66 | Male | 4 | TTT + bone cement + VSD + skin grafting | 8 | 96 | 24 | 26 | 72 | None |
| 3 | 54 | Female | 4 | TTT + bone cement + VSD + skin grafting | 20 | 126 | 32 | 18 | 91 | None |
| 4 | 64 | Male | 3 | TTT + bone cement | 7 | 24 | 21 | 30 | 61 | None |
| 5 | 49 | Male | 3 | TTT + bone cement + VSD + skin grafting | 5 | 76 | 33 | 42 | 60 | None |
| 6 | 61 | Male | 3 | TTT + bone cement | 18 | 82 | 19 | 22 | 70 | None |
| 7 | 52 | Male | 3 | TTT + bone cement | 10 | 97 | 21 | 30 | 80 | Pin-site infection |
| 8 | 49 | Female | 3 | TTT + bone cement | 6 | 48 | 17 | 41 | 81 | None |
| 9 | 56 | Male | 4 | TTT + bone cement + VSD + skin grafting | 11 | 56 | 32 | 42 | 64 | None |
| 10 | 63 | Female | 4 | TTT + bone cement + VSD + skin grafting | 9 | 20 | 38 | 32 | 72 | None |
| 11 | 50 | Male | 3 | TTT + bone cement | 25 | 87 | 20 | 23 | 82 | None |
| 12 | 64 | Male | 3 | TTT + bone cement + VSD + skin grafting | 15 | 68 | 22 | 30 | 70 | None |
| 13 | 52 | Female | 4 | TTT + bone cement + VSD + skin grafting | 8 | 75 | 39 | 45 | 74 | None |

Supplementary table 1 General information of the patients.

Supplementary table 2 T, ABI and VAS of individual patient.

| **Patient** | **Age** | **Gender** |  | T (℃) | |  | ABI | |  | VAS | |
| --- | --- | --- | --- | --- | --- | --- | --- | --- | --- | --- | --- |
|  |  |  |  | Pre-operation | 3 months post-operation |  | Pre-operation | 3 months post-operation |  | Pre-operation | 3 months post-operation |
| 1 | 45 | Male |  | 29.2 | 32.8 |  | 20 | 56 |  | 4 | 0 |
| 2 | 66 | Male |  | 28.4 | 30.4 |  | 26 | 72 |  | 6 | 1 |
| 3 | 54 | Female |  | 25.4 | 28.6 |  | 18 | 91 |  | 7 | 3 |
| 4 | 64 | Male |  | 27.5 | 30.2 |  | 30 | 61 |  | 5 | 1 |
| 5 | 49 | Male |  | 29.4 | 32.4 |  | 42 | 60 |  | 4 | 1 |
| 6 | 61 | Male |  | 24.6 | 25.2 |  | 22 | 70 |  | 3 | 0 |
| 7 | 52 | Male |  | 26.8 | 29.2 |  | 30 | 80 |  | 6 | 2 |
| 8 | 49 | Female |  | 27.2 | 29.8 |  | 41 | 81 |  | 5 | 2 |
| 9 | 56 | Male |  | 28.6 | 31.2 |  | 42 | 64 |  | 7 | 3 |
| 10 | 63 | Female |  | 26.3 | 29.8 |  | 32 | 72 |  | 5 | 2 |
| 11 | 50 | Male |  | 28.5 | 30.6 |  | 23 | 82 |  | 5 | 2 |
| 12 | 64 | Male |  | 27.4 | 29.8 |  | 30 | 70 |  | 6 | 1 |
| 13 | 52 | Female |  | 26.0 | 29.1 |  | 45 | 74 |  | 4 | 0 |
